# Exploring the use, perceptions, and challenges of mini-implants in orthodontic practice: a survey study

**DOI:** 10.3389/froh.2024.1483068

**Published:** 2025-01-07

**Authors:** Tinela Panaite, Carina Balcos, Carmen Savin, Nicolae Daniel Olteanu, Nikolaos Karvelas, Cristian Romanec, Raluca-Maria Vieriu, Alice Chehab, Irina Zetu

**Affiliations:** Department of Oral and Maxillofacial Surgery, Faculty of Dental Medicine, “Grigore T. Popa” University of Medicine and Pharmacy, Iasi, Romania

**Keywords:** professional competence, dental treatment outcome, surveys, orthodontics mini-implants perception, TAD

## Abstract

The present study aims to explore and evaluate current practices among orthodontic specialists regarding the use of mini-implants, focusing on factors influencing usage decisions, experience in placement, perceptions of outcomes, and future perspectives of these devices in orthodontic practice. It seeks to explore the association between specialists' experience levels and their perceptions of mini implants, as well as the challenges encountered in their use. It is hypothesized that orthodontic specialists' perceptions and practices regarding the usage of mini implants are influenced by various factors, including their experience, training engagement, treatment outcomes, and preferences.

**Material and methods:**

The study was conducted using an online, cross-sectional survey developed on the Survio platform to assess orthodontic specialists' perceptions of the clinical effectiveness and advantages of mini-implants in orthodontic treatments. The survey, consisting of 24 closed-ended questions in binary and multiple-choice formats, covered demographics, theoretical knowledge, clinical experience, and educational resources related to mini-implant use. Orthodontic specialists from Romania were invited to participate through the AREO association, and the survey was open for 12 weeks. Data collected from the survey were analyzed using descriptive statistics and multivariate logistic regression in SPSS software (Version 28), with a statistical significance threshold set at *p* < 0.05.

**Results:**

through a comprehensive analysis of survey data, the study investigates factors influencing specialists' perceptions, challenges encountered in practice, training engagement, material preferences, treatment outcomes, and indications for mini-implant usage. Notable correlations and discrepancies between documented literature and orthodontists' responses in Romania regarding mini-implant indications are explored, shedding light on the diversity of applications in orthodontic procedures highlighting the significance of education, training, and technical support in enhancing mini-implant utilization. Strategies to address barriers and promote informed decision-making among orthodontists are discussed.

**Conclusions:**

the study reveals diverse preferences and utilization patterns regarding mini-implants across different orthodontic procedures, reflecting the versatility and adaptability of these devices in addressing various clinical needs. By comparing documented literature with real-world practices, the study identifies both correlations and discrepancies, providing valuable insights into the practical application of mini-implants in orthodontic treatments.

## 1 Introduction

Orthodontic mini-implants, also known as mini-implants or temporary anchorage devices, are small titanium screws used as anchors in orthodontic treatment to provide additional support and control during tooth movement (1). These mini implants have gained popularity among orthodontists due to their effectiveness in cases requiring high anchorage demands (2). They are specifically designed for orthodontic purposes, being small enough to be inserted in various regions of the alveolar process, including interdental areas, without causing damage to roots (3). Orthodontic mini-implants are essential in orthodontic treatments due to their ability to provide effective anchorage in cases with high demands for stability (2). These mini-implants are typically placed transmucosally and retained enousseously, sometimes perforating both hard and soft tissues in the oral cavity (4).

The integration of mini-implants in orthodontic procedures has been shown to enhance treatment outcomes while minimizing patient compliance requirements, which is a significant advantage in clinical practice. Patient-reported outcomes related to the use of mini-implants often focus on perceived comfort, pain levels, and overall satisfaction with the orthodontic treatment process. Studies indicate that while some patients experience discomfort during the placement of mini-implants, the majority report manageable levels of pain and a high degree of satisfaction with the results achieved (5, 6).

They are particularly useful for various orthodontic tooth movements such as molar distalization, segment protraction, and rapid maxillary expansion (7). Mini-implants have been successfully utilized in treating complex malocclusions, including Class II malocclusion with anterior open bite and posterior crossbite, showcasing significant improvements in occlusion and bite closure (8). They are particularly effective in managing Class II malocclusion with severe protrusion (9). Mini-implants are widely used in orthodontics for different purposes, such as anterior intrusion, retraction to treat deep bite, and vertical maxillary excess (10). They are essential in solving anchorage problems and controlling anterior torque by varying implant positions (11).

Mini-implants have emerged as valuable adjuncts in orthodontic practice, offering enhanced treatment possibilities across a spectrum of cases. This highlights the imperative for a thorough grasp of the diverse factors impacting the efficacy and steadfastness of orthodontic mini-implants to maximize clinical effectiveness in specialized treatment. Mini-implants in orthodontics have now become an essential component of specialized care, presenting benefits such as immediate loading, multiple insertion sites, uncomplicated insertion and removal procedures, and economical advantages for patients (12). It is noteworthy that the utilization of orthodontic mini-implants has transformed orthodontic anchorage and biomechanics, ensuring impeccably steadfast anchorage (13). Moreover, assessing the oral health-related quality of life in young patients undergoing fixed orthodontic therapy is pivotal in comprehending the ramifications of orthodontic interventions on patient welfare (14).

The literature on orthodontic mini-implants reveals significant gaps regarding how specialists**'** levels of experience influence their perceptions and the challenges encountered in the application of these devices. While orthodontic mini-implants have revolutionized anchorage strategies, their successful integration into clinical practice is contingent upon the operator's expertise and familiarity with the nuances of mini-implant placement and management. Firstly, the stability of mini-implants is a critical concern that varies with the operator's experience. Research indicates that the insertion angle and the quality of cortical bone significantly affect the mechanical stability of mini-implants (15, 16). For instance, Araujo-Monsalvo et al. highlighted that the stability of mini-implants can be compromised by improper insertion angles, which may be more likely among less experienced practitioners (15). Furthermore, the torque required for removal varies with the insertion angle, suggesting that experienced clinicians may better understand how to optimize these parameters to enhance stability (16) This knowledge gap among less experienced orthodontists may lead to suboptimal outcomes, underscoring the need for targeted training and education.

The growing use of mini-implants for skeletal anchorage is becoming more apparent. Initially confined to prestigious private practices and select academic institutions, these devices have now permeated a broader array of settings. This evolution is likely propelled by compelling arguments staunchly advocating for the efficacy of orthodontic mini-implants in furnishing the coveted absolute anchorage (17–20). Mini-implants have heralded a transformation in orthodontic anchorage and biomechanics, offering a dependable alternative to traditional extraoral appliances and conferring absolute control over the anchorage, thereby mitigating undesirable side effects. Reynders et al. integrated mini-implants into orthodontic treatment planning, facilitating predictable anchorage control and enhancing the ability to rectify severe skeletal and dental discrepancies (21). Several investigations have consistently pointed out the lack of widespread use of orthodontic mini-implants in clinical settings (22). To delve into this issue of implementation, we conducted a survey-based study to uncover obstacles hindering the adoption of orthodontic mini-implants. This study seeks to explore the perspectives, difficulties, and procedures concerning the adoption of mini-implants in orthodontic practice among specialists. This study aims to examine how orthodontic specialists**'** levels of experience relate to their views on mini-implants and the obstacles faced in their application. It is hypothesized that orthodontic specialists**'** perceptions and practices regarding the usage of mini-implants are influenced by various factors, including their experience, gender, training engagement, type of practice (e.g., private vs. public), availability of resources, and patient demographics. These variables may result in differing outcomes in terms of mini-implant usage, challenges encountered, and the perceived benefits of incorporating these devices into orthodontic treatments.

## 2 Materials and methods

### 2.1 Ethical clearance

Approval for this study was obtained from the Research Ethics Committee of Grigore T. Popa University of Medicine and Pharmacy Iasi, under No. 178/02.05.2022. The Committee also approved the participant information and consent forms used in the research.

### 2.2 Study framework

This study adopted an online cross-sectional survey approach. Cross-sectional studies, also referred to as prevalence studies, involve observing selected variables from a sample population within a defined timeframe.

### 2.3 Questionnaire development and validation

The online survey was developed on the Survio platform to assess orthodontic specialists' perceptions of the clinical effectiveness and advantages of orthodontic mini-implants in specialty treatment. The questionnaire was designed to cover demographics, theoretical knowledge, clinical experiences, and educational resources. It consisted of 24 questions in binary and multiple-choice formats.

To ensure the validity of the questionnaire, the following steps were undertaken:

- Content validity: the questionnaire was reviewed by a panel of experts in orthodontics and research methodology to ensure that the questions comprehensively covered the relevant topics. Adjustments were made based on their feedback to improve clarity and relevance.
- Pilot testing: a pilot study was conducted with a small sample of orthodontic specialists (*n* = 30) to test the clarity, coherence, and length of the questionnaire. The feedback received was used to refine the wording of certain questions and eliminate any ambiguous or unclear items.

The final version of the questionnaire was approved for use in the main study.

### 2.4 Online survey

The online survey was developed on the Survio platform to assess orthodontic specialists' perceptions of the clinical effectiveness and advantages of orthodontic mini-implants in specialty treatment. The questionnaire covered demographics, theoretical knowledge, clinical experiences, and educational resources. The survey consisted of 24 questions in binary and multiple-choice formats. The aim of the present study is to explore and evaluate current practices among orthodontic specialists regarding the use of mini-implants, with a focus on factors influencing usage decisions, experience in placement, perceptions of outcomes, and future perspectives of these devices in orthodontic practice.

Employing a qualitative research design, the study utilized surveys to collect data from orthodontic practitioners. Participants were questioned about their experiences, opinions, and observations concerning clinical outcomes and the benefits associated with incorporating mini-implants into orthodontic treatments. The questionnaire comprised an introductory section providing details about the investigator, research purpose, and confidentiality agreement, followed by identification questions pertaining to participants' socio-demographic characteristics. Additionally, the questionnaire included 24 opinion-based questions with both single-choice and multiple-choice response options, formulated in a closed-ended manner.

### 2.5 Survey distribution

Orthodontic specialists in Romania were invited to participate in the survey through the Association AREO. The survey remained open for 12 weeks, with reminders sent at two and six weeks to non-respondents.

### 2.6 Data collection

Survio platform was utilized for questionnaire administration. No personally identifiable data was gathered, and the data access was restricted solely to the principal investigator (T.P.). Moreover, Internet Protocol (IP) addresses were not logged.

### 2.7 Data analysis

Upon questionnaire completion, the collected data was inputted into a Microsoft Excel spreadsheet and subsequently imported into SPSS software (Version 28, IBM Corp., Armonk, New York) for statistical analysis. The analysis process involved: Initially establishing a database for statistical examination. Conducting descriptive analysis, which encompassed calculating means, medians, confidence intervals, and percentages; Employing qualitative variable analysis methods such as the Pearson Chi-square test (for parametric cases), performing a comparison between qualitative and quantitative variables utilizing the Compare Means test, coupled with ANOVA testing to ascertain statistical significanceMultivariate logistic regression was used to identify the factors that influence participants' attitudes towards the use of implants in treatments. The statistical significance threshold for establishing a relationship as significant was set at a probability value of *p* < 0.05.

### 2.8 Data availability

The data collected in this study are stored on the Survio platform and are accessible only to the principal investigator. Due to confidentiality constraints, the datasets are not publicly available. However, they can be provided upon reasonable request, in compliance with data protection regulations.

## 3 Results

The study sample consisted of 159 dental specialists in orthodontics from the North-East region of Romania. Out of a total of 250 orthodontists invited to participate in the study, 159 completed the survey. This represents a response rate of 63.6%. Of the total participants, 76.7% were female, and 49.7% were in the age group of 30–40 years old. More than half of the participants had less than 5 years of professional experience in the field of orthodontics, and 96.2% of them practiced both in rural and urban areas (Table 1).

**Table 1:** Demographic characteristics of the study sample.

Variables	No	%
Age		
<30 years old	46	28.9%
30–40 years old	79	49.7%
41–50 years old	20	12.6%
>50 years old	14	8.8%
Gender		
Female	122	76.7%
Male	37	23.3%
Work experience as orthodontist		
<5 years	84	52.8%
5–10 years	45	28.3%
11–20 years	18	11.3%
>20 years	12	7.5%
Work area		
Rural area	6	3.8%
Urban and rural area	153	96.2%

Evaluating the reasons behind orthodontists' use of mini-implants shows that “The possibility of offering more efficient treatments” (93.7%) is the primary reason, especially for male doctors with less than 5 years of experience (98.8%, *p* = 0.045). However, there are also situations where the placement of mini-implants is performed by surgeons, particularly among doctors who feel they are not adequately prepared (43.4%). This situation is more commonly reported by female participants with limited experience (less than 5 years, 53.6%) (Table 2).

**Table 2:** Motivation and perspectives on the use of mini-implants by gender and work experience.

		Gender			Work experiences as orthodontist				
	Yes%	Female	Male	*p*	<5 years	5–10 years	11–20 years	>20 years	*p*
What reasons or circumstances led you to start placing orthodontic mini-implants?									
Increased patient demand	4.4	4.1%	5.4%	.599	1.2%	6.7%	11.1%	8.3%	.045*
Possibility of offering more efficient treatments	93.7	93.4%	94.6%		98.8%	86.7%	88.9%	91.7%	
Other reasons (please specify)	1.9	2.5%	0.0%		0.0%	6.7%	0.0%	0.0%	
What factors influence your choice between personally placing orthodontic mini-implants and referring patients to surgeons?									
Need for local anesthesia administration	8.2	9.8%	2.7%	.268	7.1%	13.3%	5.6%	0.0%	.364
Longer treatment duration in the dental office	10.7	9.8%	13.5%		10.7%	15.6%	0.0%	8.3%	
Potential to manage acute pain	10.7	9.8%	13.5%		10.7%	11.1%	11.1%	8.3%	
Lack of proper training or instruction	43.4	46.7%	32.4%		53.6%	28.9%	38.9%	33.3%	
Other reasons (please specify)	39	32.0%	62.2%		31.0%	40.0%	55.6%	66.7%	
How many practical or theoretical courses have you attended related to the use of orthodontic mini-implants?									
More than 5 practical courses with live demonstrations	13.2	11.5%	18.9%	.165	9.5%	15.6%	11.1%	33.3%	.288
1–5 practical courses with live demonstrations	44.0	41.0%	54.1%		40.5%	44.4%	55.6%	50.0%	
1–5 theoretical courses	34.0	37.7%	21.6%		38.1%	31.1%	33.3%	16.7%	
I have not attended any specialized courses	8.8	9.8%	5.4%		11.9%	8.9%	0.0%	0.0%	
What is your perspective on the future use of orthodontic mini-implants in orthodontic practice?									
Increased use	81.8	87.7%	59.5%	.103	88.1%	66.7%	88.9%	75.0%	.188
Moderate use	17.0	12.3%	32.4%		10.7%	26.7%	16.7%	25.0%	
Decreased use	4.4	3.3%	8.1%		6.0%	4.4%	0.0%	0.0%	
Not sure	2.5	1.6%	5.4%		0.0%	8.9%	0.0%	0.0%	

* Test Anova, significant at a *p* = 0.050.

Thorough training for this treatment is essential, as nearly 80% of participants attended 1–5 courses with or without practical demonstrations. Those with less than 5 years of practice (38.1%) mostly attended theoretical courses, while participants with more than 11 years of experience attended courses that included practical components (55.6%) (Table 2).

Regarding the participants' perspective on the future use of these mini-implants, more than 80% believe that they will be used more frequently, with the majority being female participants (87.7%). The increase in the use of these mini-implants is primarily declared by participants with up to 5 years of experience and those with 11–20 years of experience in this activity (88.1% and 88.9%, respectively) (Table 2).

Table 3 presents the results obtained from evaluating the knowledge and attitudes regarding the use of mini-implants by gender and years of experience. More than 60% of participants prefer to use titanium implants, with a higher preference among female participants (69.7%) and those with less than 5 years of experience (69%). The indications for using these implants are diverse in orthodontics. They range from the intrusion of extruded teeth (61.6%), indirect anchorage for space closure (45.9%), intrusion to resolve open bites (44.0%), molar uprighting (42.4%), molar mesialization (37.1%), en masse retraction of the anterior group (35.8%), and intrusion to resolve occlusal cant (30.2%).

**Table 3:** Cunostinte si atitudini privind utilizarea mini-implantelor gender si work experience.

		Gender			Work experiences as orthodontist				
	Yes%	Female	Male	*p*	<5 years	5–10 years	11–20 years	>20 years	*p*
What materials do you prefer for orthodontic mini-implants (e.g., titanium, zirconia)?									
Titanium	65.4	69.7%	51.4%	.119	69.0%	64.4%	61.1%	50.0%	.636
Stainless steel	6.3	5.7%	8.1%		7.1%	6.7%	5.6%	0.0%	
No preference	28.3	24.6%	40.5%		23.8%	28.9%	33.3%	50.0%	
What is your main reason for using orthodontic mini-implants?									
Mesialization of molars	37.1	36.1%	40.5%	.236	31.0%	35.6%	61.1%	50.0%	.342
Indirect anchorage for space closure	45.9	50.8%	29.7%		40.5%	51.1%	50.0%	58.3%	
Intrusion of extruded teeth	61.6	62.3%	59.5%		63.1%	62.2%	61.1%	50.0%	
Intrusion for resolving open bite	44.0	40.2%	56.8%		50.0%	31.1%	44.4%	50.0%	
Uprighting molar	42.4	45.9%	35.1%		45.2%	35.6%	61.1%	33.3%	
Retraction en masse of the frontal group	35.8	31.1%	51.4%		45.2%	17.8%	38.9%	33.3%	
Intrusion for resolving occlusal plane cant	30.2	24.6%	48.6%		32.1%	28.9%	22.2%	33.3%	
Distalization of molars	41.5	38.5%	51.4%		48.8%	28.9%	33.3%	50%	
Traction of impacted teeth	23.3	25.4%	1.2%		29.8%	24.4%	5.6%	0.0%	
Attachment for facial mask	2.5	1.6%	5.4%		3.6%	0.0%	5.6%	0.0%	
What are the main advantages of using orthodontic mini-implants?									
Increased efficiency of orthodontic treatments	91.2	91.0%	91.9%	.312	94.0%	82.2%	94.4%	100.0%	.206
Reduced need for dental extractions	22.6	19.7%	32.4%		21.4%	26.7%	11.1%	33.3%	
Fewer side effects compared to other methods	43.4	47.5%	29.7%		53.6%	35.6%	33.3%	16.7%	
Faster recovery for patients	8.8	9.8%	5.4%		13.1%	0.0%	16.7%	0.0%	
Reduced treatment time	55.3	60.7%	37.8%		65.5%	48.9%	33.3%	41.7%	
Others (please specify)	1,6	1.6	0.0%		2.4%	0.0%	0.0%	0.0%	
What are the main disadvantages of using orthodontic mini-implants?									
Increased risk of infections or complications	22.0	21.3%	24.3%	.415	21.4%	28.9%	22.2%	0.0%	.199
Higher costs for patients	72.3	76.2%	59.5%		79.8%	60.0%	83.3%	50.0%	
Difficulty in maintaining oral hygiene	35.8	38.5%	27.0%		33.3%	37.8%	27.8%	58.3%	
Need to remove the mini-screws at the end of treatment	5.0	2.5%	13.5%		3.6%	8.9%	5.6%	0.0%	
Possible discomfort or pain for patients	59.7	59.8%	59.5%		70.2%	55.6%	38.9%	33.3%	
Others (please specify)	7.5	8.2%	5.4%		11.9%	4.4%	0.0%	0.0%	
How do you evaluate the results of orthodontic treatments using mini-implants?									
Satisfactory	56.6	54.1%	64.9%	.142	54.8%	68.9%	44.4%	41.7%	.094
Very satisfactory	41.5	44.3%	32.4%		45.2%	24.4%	55.6%	58.3%	
Unsatisfactory	1.3	1.6%	0.0%		0.0%	4.4%	0.0%	0.0%	
Very unsatisfactory	0.6	0.0%	2.7%		0.0%	2.2%	0.0%	0.0%	
Have you observed a significant difference in the results of orthodontic treatments using mini-implants compared to traditional treatments?									
Yes	81.1	81.1%	81.1%	.939	84.5%	66.7%	94.4%	91.7%	.021*
No	5.7	5.7%	5.4%		2.4%	8.9%	11.1%	8.3%	
Not sure	14.5	13.9%	16.2%		13.1%	24.4%	5.6%	0.0%	
How do you evaluate the immediate loading of mini-implants in orthodontic treatments?									
I always practice it	39.0	38.5%	40.5%	.826	38.1%	42.2%	33.3%	41.7%	.724
I frequently practice it	33.3	32.8%	35.1%		35.7%	28.9%	38.9%	25.0%	
I rarely practice it	19.5	19.7%	18.9%		21.4%	17.8%	11.1%	25.0%	
I do not practice it at all	10.1	9.8%	10.8%		6.0%	11.1%	27.8%	8.3%	

The advantages selected by the study participants include “Increasing the effectiveness of orthodontic treatments” (91.2%), “Fewer side effects compared to other methods” (43.4%), and “Reducing the time required for treatment” (55.3%), with similar distribution of opinions across both genders and years of experience.

The disadvantages frequently selected by participants are “Higher costs for patients” (72.3%), “Difficulties in maintaining dental hygiene” (35.8%), and “Possible discomfort or pain for patients” (59.7%). Despite these disadvantages, more than 90% of participants who use mini-implants consider the treatment outcomes to be “satisfactory” (56.6%) and “very satisfactory” (41.5%), especially since 81.1% of participants observed significant differences in the treatment results achieved with mini-implants compared to traditional orthodontic treatment. Participants with over 11 years of experience are more likely to perceive a significant difference between the two types of treatments compared to those with less experience (*p* = 0.021).

When questioned about possible challenges or failures related to the use of mini-implants, the results of the statistical analysis show that 52.2% of participants prefer to use a new implant instead of reusing one that failed, while 43.2% prefer to reuse it. This attitude was identical in the gender distribution, where the difference recorded was statistically significant (*p* = 0.046), as well as in the distribution by years of experience. The most frequent biological, mechanical, or iatrogenic complications were screw loosening (83%), soft-tissue overgrowth/irritation (59.7%), and irritation caused by auxiliary springs (31.4%).

Side effects were reported by less than half of the participants, with 25.8% reporting excessive torquing/tipping of teeth as a common side effect, 17% reporting excessive extrusion/intrusion of teeth, and 19% reporting root resorption. The biggest challenge regarding the use of mini-implants seems to be the lack of proper training or instruction (45.3%), with female participants and those with less than 5 years of experience choosing this option most frequently (49.2% and 57.1%, respectively) (Table 4).

**Table 4:** Provocari, complicatii si esecuri in utilizarea mini implantelor.

		Gender			Work experiences as orthodontist				
	Yes%	Female	Male	*p*	<5 years	5–10 years	11–20 years	>20 years	*p*
When faced with a failed mini-implant, what do you prefer to do?									
Sterilize the mini-implant and insert it in another location	45.3	45.9%	43.2%	.046*	45.2%	44.4%	44.4%	50.0%	.956
Replace with another mini-implant	52.2	53.3%	48.6%		52.4%	51.1%	55.6%	50.0%	
Abandon the idea of treatment with mini-implants	2.5	0.8%	8.1%		2.4%	4.4%	0.0%	0.0%	
What are the most frequent biological, mechanical, or iatrogenic complications?									
Screw loosening	83.0	82.0%	86.5%	.523	82.1%	82.2%	77.8%	100.0%	.412
Soft-tissue overgrowth/irritation	59.7	60.7%	56.8%		59.5%	64.4%	83.3%	8.3%	
Irritation caused by auxiliary springs	31.4	31.1%	32.4%		31.0%	26.7%	38.9%	41.7%	
Sinus perforation	4.4	4.9%	2.7%		8.3%	0.0%	0.0%	0.0%	
Subcutaneous emphysema	5.0	3.3%	10.8%		4.8%	8.9%	0.0%	0.0%	
Others	1.6	1.6%	0.0%		2.4%	0.0%	0.0%	0.0%	
Which of the following side effects have been associated with the use of orthodontic mini-implants in your treatments?									
Excessive torquing/tipping of teeth	25.8	25.4%	27.0%	.270	29.8%	26.7%	11.1%	16.7%	.253
Excessive extrusion/intrusion of teeth	17	13.9%	27.0%		14.3%	22.2%	16.7%	16.7%	
Root resorption	11.9	8.2%	24.3%		8.3%	22.2%	5.6%	8.3%	
I have not observed any side effects	58.5	61.5%	48.6%		58.3%	44.4%	83.3%	75.0%	
What are the main challenges in using mini-implants?									
Administration of local anesthesia	11.3	8.2%	21.6%	.217	7.1%	22.2%	11.1%	0.0%	.257
Longer treatment duration in the dental office	15.7	16.4%	13.5%		10.7%	17.8%	22.2%	33.3%	
Management of acute pain	15.7	12.3%	27.0%		15.5%	17.8%	22.2%	0.0%	
Lack of proper training or instruction	45.3	49.2%	32.4%		57.1%	31.1%	33.3%	33.3%	
Others	23.3	21.3%	29.7%		21.4%	26.7%	16.7%	33.3%	
How do you rate the level of anxiety in patients undergoing this treatment?									
Mild	27.7	28.7%	24.3%	.700	25.0%	28.9%	27.8%	41.7%	.652
Moderate	64.8	63.1%	70.3%		65.5%	62.2%	72.2%	58.3%	
Very high	7.5	8.2%	5.4%		9.5%	8.9%	0.0%	0.0%	

* Test ANOVA, significant at *p* = 0.05.

During the application of implants, the patient also plays a decisive role, as anxiety and age can make this type of treatment difficult. 64.8% of participants consider patients undergoing this treatment to have a moderate level of anxiety (Table 4).

Multiple regression analysis of the demographic variables (gender, years of practice in the specialty) was performed to determine the predictors (Table 5).

**Table 5:** Multiple regression analysis of predictors for factors that influence their choice between personally placing orthodontic mini-implants and referring patients to surgeonsvs.

					95% Confidence interval for Exp (B)	
	B	Srd. error	Sig	OR(*β*)	Lower bound	Upper bound
Need for local anesthesia administration						
Gender (female)	−1.342	1.061	.206	.261	.033	2.090
Years of experience as an orthodontist	−.115	.335	.731	.891	.462	1.720
Longer treatment duration in the dental office						
Gender (female)	.412	.574	.473	1.510	.490	4.653
Years of experience as an orthodontist	−.230	.305	.450	.794	.437	1.444
Potential to manage acute pain						
Gender (female)	.373	.573	.515	1.452	.473	4.464
Years of experience as an orthodontist	−.059	.282	.835	.943	.543	1.638
Lack of Proper Training or Instruction						
Gender (female)	−.537	.401	.180	.585	.267	1.282
Years of experience as an orthodontist	−.342	.185	.064	.710	.495	1.020
Continuing Education Courses						
1–5 practical courses with live demonstrations						
Gender (female)	−.178	.538	.741	.837	.292	2.401
Years of experience as an orthodontist	−.250	.241	.300	.779	.485	1.249
1–5 theoretical courses						
Gender (female)	−.969	.610	.112	.379	.115	1.253
Years of experience as an orthodontist	−.514	.268	.055	.598	.354	1.011
No specialized courses attended						
Gender (female)	−.941	.910	.301	.390	.066	2.321
Years of experience as an orthodontist	−1.138	.520	.029	.321	.116	.889

Demographic characteristics(gender. years of practice).

In the case of the need for anesthesia administration, female subjects as well as those with more years of experience are not predictors for personally placing dental implants (*β* = 0.261, *p* = 0.261 and *β* = 0.891, *p* = 0.731 respectively). A long duration of traditional treatment is another factor that can motivate orthodontists to apply implants. Thus, female subjects can be a predictor for the use of implants for this reason (*p* = 0.473, *β*=1.510), while more experienced specialists are not predictors (*p* = 0.731, *β* = 0.891). The same situation is encountered in the potential to manage acute pain, for which female subjects can be a predictor for the use of implants (*p* = 0.515, *β*= 1.450), while more experienced specialists are not (*p* = 0.835, *β* = 0.943). Lack of proper training or instruction remains a serious reason for not applying implants for both demographic variables analyzed.

Regarding the level of training, female subjects as well as those with more years of experience are not predictors for attending continuing education courses for the use of implants.

## 4 Discussion

The findings from our research align closely with those of two extensive surveys previously conducted within the orthodontic field regarding practitioners' encounters with mini-screws (23–27). Our results align with previous surveys conducted within the orthodontic field regarding practitioners' experiences with mini-screws. Factors such as insufficient training, apprehensions about complications, and specific clinical prerequisites significantly impact the decision of orthodontists to use mini-implants in their practice.

### 4.1 Training and education

The lack of adequate preparation and education on orthodontic mini-implant usage can greatly affect their efficacy and stability. Proper training is essential for practitioners to adeptly select, position, and manage orthodontic mini-implants, considering variables like patient demographics, implant characteristics, and post-implantation care. Fatani et al. (26) highlighted that insufficient education and training are major barriers to the adoption of mini-implants among orthodontists in Saudi Arabia. Similarly, Ananthanarayanan et al. (27) noted that despite the biocompatibility and corrosion resistance of orthodontic mini-implants, inadequate resistance to fracture under significant orthodontic loads remains a concern, emphasizing the need for better training in selecting suitable mini-implants for various clinical scenarios.

### 4.2 Impact of experience and gender

Our multiple regression analysis revealed that neither gender nor years of experience significantly predict the likelihood of orthodontists personally placing dental implants due to the need for anesthesia administration. However, female orthodontists are more likely to be influenced by the long duration of traditional treatments and the potential to manage acute pain, unlike their more experienced counterparts. This suggests targeted training might be beneficial in addressing specific concerns among different demographic groups.

### 4.3 Clinical advantages and challenges

Orthodontic mini-implants offer several clinical advantages such as improved anchorage, simplified surgical techniques, versatile applications, and predictable treatment outcomes. These benefits contribute to increased treatment efficiency and minimally invasive, patient-centered approaches. However, complications related to soft tissues, such as inflammation and infections, particularly in the palatal area, remain a significant concern. These issues highlight the need for ongoing education and training to mitigate complications and improve outcomes.

### 4.4 Materials and preferences

Despite a preference for titanium among respondents, the literature indicates that the material of mini-implants, whether steel or titanium, does not significantly impact their success rate. Stainless steel, with its lower cost and similar clinical efficiency, is a viable option, underscoring the importance of material selection based on clinical requirements and cost-effectiveness rather than personal preference alone.

The proportion of orthodontists who opt not to utilize mini-implants in their practice is a topic of significant interest due to its ramifications for treatment outcomes. The variable “Lack of preparation or adequate education” in the context of orthodontic mini-implant usage has been thoroughly investigated, yielding valuable insights. Furthermore, the deficiency in preparation and education has been identified as a primary driver of the underutilization of orthodontic mini-implants (28, 29). This underscores the pivotal role of comprehensive educational and training programs in fostering the adoption and effective utilization of mini-implants in orthodontic practice.

One of the frequently reported complications in this analysis was related to the soft tissues around the implant. Placing mini-implants can stimulate surrounding soft tissues and trigger tissue inflammation, infections, and peri-implantitis, especially when placed in mobile mucosa. Excessive tissue development, defined as partial or total coverage of the implant head by surrounding soft tissues, has been reported by Ruiz et al. as the most common complication associated with mini-implants placed in the palatal area (30). Traumatic injuries to soft tissue and soft tissue coverage can occur in the form of aphthous ulcers or wounds at the level of the alveolar, buccal, labial, or frenulum mucosa. Inflammation near the mini-implant occurs in the palate, buccal fold, and ascending ramus. In patients with poor oral hygiene, inflammation may occur even when the placement procedure is performed carefully.

The decision to start using orthodontic mini-implants in practice can be influenced by various factors and circumstances. Some of the main reasons or circumstances that may motivate practitioners to begin using orthodontic mini-implants include:

a. Simplification and effectiveness of orthodontic treatments: the use of mini-implants has simplified and improved the effectiveness of many orthodontic treatments, reducing unwanted tooth movements, especially in adult patients (31).
b. Improved anchorage and treatment options: mini-implants provide improved anchorage for force application, allowing a wider range of treatment options and enhancing treatment outcomes (32).
c. Addressing specific clinical challenges: mini-implants offer solutions for specific clinical challenges, such as maxillary molar intrusion, Class II skeletal malocclusion, and severe Class II division 2 malocclusion, providing orthodontists with effective tools for addressing complex cases (33).
d. Enhanced treatment efficiency: the use of mini-implants has led to increased treatment efficiency, allowing procedures such as en masse retraction of maxillary anterior teeth and skeletal expansion appliances with increased precision (34, 35).
e. Minimally invasive and patient-centered approaches: mini-implants provide minimally invasive options for orthodontic anchorage, allowing patient-centered approaches and reducing the need for more invasive procedures (36).

Orthodontic mini-implants offer several advantages compared to other orthodontic treatment options. The main advantages:

a. Improved anchorage: mini-implants provide stable and reliable anchorage, allowing the application of orthodontic forces without depending on patient compliance, thereby expanding treatment options in orthodontics (37).
b. Simplified surgical technique: the simplicity of the surgical technique for mini-implant placement, along with minimal patient stress and a favorable cost-benefit ratio, makes them an attractive option in orthodontic practice (38).
c. Versatile applications: mini-implants can be used for various orthodontic applications, including en masse retraction, Class II skeletal malocclusion correction, maxillary skeletal expansion, maxillary incisor intrusion, open bite treatment, and orthodontic space closure, providing versatile treatment options (39).
d. Predictable treatment outcomes: mini-implants offer predictable treatment outcomes, with high success rates similar to regular-sized implants in single-tooth replacement cases, providing reliable solutions for orthodontic treatment (40).
e. Minimal patient stress: mini-implants offer simple, atraumatic procedures for insertion and removal, minimal patient stress, and a favorable cost-benefit ratio, contributing to patient comfort and treatment efficiency (40).
f. Stability and success: mini-implants ensure stable anchorage and have been proven effective in asymmetric tooth movements, intrusion mechanics, and intermaxillary fixation/traction, contributing to treatment success (41).
g. Bone adaptation and healing: mini-implants should be left in the placement site for a healing period to allow bone adaptation and increase the success rate, especially in adolescent patients (41).

These advantages make orthodontic mini-implants a valuable and versatile tool in orthodontic practice, offering predictable treatment outcomes, improved anchorage, and minimal patient stress. There is a correlation between specialized literature (41) and Romanian orthodontists' responses regarding the assessment of immediate loading of mini-implants in orthodontic treatments.

### 4.5 Study limitations

a. This study is based on a self-reported survey, which carries the risk of two specific biases:
  - social desirability bias: participants may provide answers that they believe will be viewed favorably by others rather than their actual behaviors or opinions.
  - recall bias: because the survey requires participants to reflect on past experiences with mini-implants, there is a possibility that their recollections may be incomplete or inaccurate.
b. The study uses a cross-sectional design, which captures data from respondents at a single point in time. While this design allows us to explore associations between variables, such as experience level and perceptions of mini-implants, it limits our ability to establish causal relationships.
c. Sample size and diversity consisted of 159 dental specialists from the north-east region of Romania.
d. Non-response Bias: the survey had a response rate of 63.6%, meaning that a significant portion of the invited participants did not respond. There is a possibility that the views and practices of non-respondents may differ from those who chose to participate, potentially affecting the generalizability of the results.
e. Limited geographic representation: the study was conducted among orthodontists from a specific region of Romania (the north-east), which may not reflect the practices and perspectives of orthodontists in other parts of the country or globally. Thus, the findings may not be fully generalizable to a broader population of orthodontic specialists.
f. Limited exploration of confounding factors:the study primarily focused on variables such as experience level and perceptions of mini-implants but did not extensively explore other potential confounding factors, such as access to resources, the specific nature of clinical cases, or the availability of advanced training programs, which could also influence the use of mini-implants.
g. Questionnaire validation and adaptation: although steps were taken to validate the questionnaire through expert review and pilot testing, cultural or regional differences in the understanding of certain questions may still exist. These subtle differences may have influenced how participants interpreted and responded to specific survey items.

### 4.6 Implications for future research and clinical practice

a. *Training and education*: one of the most significant findings is the role that insufficient training plays in the hesitation of orthodontists to adopt mini-implants, especially among less experienced practitioners. This highlights the need for more comprehensive and standardized training programs, including both theoretical and practical components.
b. *Broader adoption of mini-implants*: future research should investigate how collaborative care models (between orthodontists and surgeons) could be optimized and whether additional training could enable more orthodontists to handle mini-implant placements independently.
c. Geographic and demographic differences: given the limited geographic scope of this study, it would be valuable to replicate similar studies in other regions, both within and outside Romania, to understand how cultural, regional, and resource-related factors impact mini-implant usage. Comparative international studies could help identify best practices that are effective across different healthcare systems, potentially informing global clinical guidelines.
d. Addressing patient concerns: the findings also point to patient-related challenges, such as anxiety and discomfort. Future clinical trials could focus on strategies for managing patient anxiety and improving comfort during mini-implant placement. This might include advancements in minimally invasive techniques, improved anesthesia options, or better patient education on the benefits and risks of mini-implants.
e. *Advancements in mini-implant design and materials*: the preference for titanium mini-implants in this study suggests a potential area for future research into new materials and designs that could offer similar or improved clinical efficacy at a lower cost. Studies comparing different materials or implant designs in terms of success rates, patient comfort, and cost-effectiveness could offer valuable insights for improving clinical practice.

## 5 Conclusions

Our study highlights both unique and shared perspectives among Romanian orthodontists regarding the use of mini-implants. As hypothesized, orthodontic specialists' perceptions and practices regarding mini-implant usage were influenced by various factors, including their level of experience, gender, and training engagement. The primary motivation for using mini-implants was the potential for more efficient treatments, particularly among male practitioners with less than five years of experience. In contrast, many female practitioners with limited experience preferred referring implant placement to surgeons due to feeling inadequately prepared. Over 80% of participants anticipate an increase in mini-implant use, especially among female orthodontists and those with either less than five years or between 11 and 20 years of experience. These findings support the hypothesis that differences in training and experience levels result in varying practices and perceptions of mini-implant use. This underscores the need for ongoing education and tailored training programs to bridge confidence and skill gaps, ensuring all practitioners can effectively utilize mini-implants in their treatments. Future research could expand to other regions or countries to provide a more comprehensive understanding of global practices and variations in mini-implant use. Exploring the influence of advanced training programs on mini-implant success rates and practitioner confidence over time would further enrich the field of orthodontic practice.

## Data Availability

Publicly available datasets were analyzed in this study. This data can be found here: Tinela Panaite, tinela-panaite@umfiasi.ro.
